# Expression of NEAT1 can be used as a predictor for Dex resistance in multiple myeloma patients

**DOI:** 10.1186/s12885-023-11084-x

**Published:** 2023-07-05

**Authors:** Yuyue Ren, Yijun Liu, Wanting He, Weiwei Zhao, Jiaqi Pan, Haiyan Gao, Yuying Li, Ying Zhang, Wei Wang

**Affiliations:** 1 The Second Affiliated Hospital of Harbin Medical University, No.246 Xuefu Street Nangang Block, 150081 Harbin, Heilongjiang Province P.R. of China; 2 Yanda Lu Daopei Hospital, Yanjiao Economic Development Zone, 101118 Sanhe, Langfang, Hebei Province P.R. of China

**Keywords:** Multiple myeloma, lncRNA, NEAT1, Dex resistantce, Diagnosis

## Abstract

**Objective:**

Multiple myeloma is a heterogeneous disorder and the intratumor genetic heterogeneity contributes to emergency of drug resistance. Dexamethasone has been used clinically for decades for MM. Nevertheless, their use is severely hampered by the risk of developing side effects and the occurrence of Dex resistance. LncRNA NEAT1 plays a oncogenic role and participates in drug resistance in many solid tumors. Therefore, we investigated a potential usefulness of this molecular as a biomarker for diagnosis of MM and possible correlations of NEAT1 expression with drug resistance and prognosis.

**Methods:**

Bone marrow and peripheral blood mononuclear cells samples were collected from 60 newly diagnosed MM patients. The expression of NEAT1expression level were detected by quantitative real-time PCR analyses. The relationship about the expression levels of lncRNA with other clinical and cytogenetic features was analyzed. In addition, we measured to analysis the correlation between the expression of NEAT1 and Dex resistance in MM patients.

**Results:**

It was found that the expression of NEAT1 is significantly higher in multiple myeloma patients compared to controls and does not change with other clinical features and cytogenetic features. We further discovered that overexpression of NEAT1 was associated with Dex resistance and a poor prognosis in MM patients.

**Conclusion:**

LncRNA NEAT1 has a significant value that might act as a promoting factor in the development of MM and may be severed as a diagnostic factor in MM. NEAT1 invovled in Dex resistance, which provide a new interpretation during the chemotherapy for MM.

**Supplementary Information:**

The online version contains supplementary material available at 10.1186/s12885-023-11084-x.

## Introduction

Multiple myeloma (MM) is a B cell neoplasm characterized by aberrant prolifesration of monoclonal plasma cell in bone marrow, leading to various endougan damage [1], including anemia and other cytopenias, bone lesions renal dysfunction, compromised immune function, and peripheral neuropathy. It is reported that the survival time of patients with MM varies from a few weeks to more than 10 years [2]. With the availability of agents such as thalidomid and bortezomib, the overall survival has extended recently, but Dexamethasone (Dex) is still a key front-line chemotherapeutic for B-cell malignant MM, participating in multi-drug chemotherapy regimens [3]. However, Dex resistance is inevitable, even affects the prognosis and threatens the life of patient [4, 5]. Therefore, efficacious approaches identifying Dex resistant of MM patients are essential for developing new effective therapeutic targets, improving the prognostic situation and extending the survival time of MM.

Nowadays, as new therapeutic methods quickly progress, more precise diagnostic methods was needed to better stratify patients. However, it is difficult to correctly evaluate the efficacy of treatment except for the informative examination of BM smears. Myeloma protein(M protein) is the most common means of detection, but the detection of M protein had a plateau, and the sensitivity of peripheral blood(PB) M protein level detection to monitor the therapeutic effect still needs to be improved. Some studies have suggest that specific chromosomal abnormalities may influenced the outcome of MM patients. Previous study have demonstrated that the loss of chromosomes 13 and 17p (del 13 and del17p), observed in many MM patients, has a negative impact on both EFS and OS, but chromosome tests are still inconvenient, expensive, and require a long-time running [6]. Therefore, there is still a lack of convenient and sensitive biological factors.

Long non-coding RNAs (lncRNA) is a vast class of non-protein coding transcripts that is longer than 200 bp [7]. Increasing evidence has been found that lncRNA, as a cancer hall-marks, may play a role via discrete modules that decoy, guide, or scaffold other regulator proteins involving proliferation, apoptosis, metastasis, metabolism, senescence and drug-resistance [8, 9]. For example, lncRNA MALAT1 promotes cell proliferation in lung adenocarcinoma [10]. In the breast cancer, lncRNA NKILA is a negative feedback regulator and suppresses cancer metastasis [11]. LncRNA XIST exerts tumor-suppressive functions by up-regulating miR-152 glioblastama stem cells [12]. HOTAIR servers as a prognostic factor for colorectal cancer [13]. LncRNA-TUSC7/miR-224 affected chemotherapy resistance of esophageal squamous cell carcinoma [14]. For multiple myeloma, recent studies have also shown that lncRNAs are overexpressed in patients with MM compared to healthy individuals [15, 16]. Although accumulating evidence indicates that lncRNA severed as vital regulators involved in diverse aspects of gene regulation at transcriptional, posttranscriptional and epigenetic levels, and participate in a variety of biological processes, only a few number of lncRNAs have been characterized functionally [8, 17].

The nuclear-enriched abundant transcript 1 (NEAT1) which located on chromosome 11, is a kind of lncRNAs, and it was biologically well-studied [18]. It plays an critical carcinogenic role in promoting tumorigenesis of various human cancers, and previous study has shown that high expression of NEAT1 is associated with worse outcome in many kinds of cancer [19], such as esophageal squamous cell carcinoma [20], colorectal cancer [21], lung cancer [22], ovarian cancer [23], prostate cancer [24], as well as hematological malignancy [25]. Other than, lncRNA NEAT1 participate in several biological processes, also involved in drug resistance. In gastric cancer, for instance, researchers found that silence of lncRNA NEAT1 inhibits malignant biological behaviors and therapy resistance [26, 27].In breast cancer, the down-regulation of NEAT1 increased cancer cells chemo-sensitivity [28]. But in leukemia, researchers found that the overexpression of lncRNA NEAT1 can revers drug resistance through the inhibition of ABCG2 [29]. So that, NEAT1 plays a complicated role in drug resistance of different tumors. However, the clinical significance of lncRNA NEAT1 in MM remains unclear.

In current study, we identified that the expression level of lncRNA NEAT1 was increased in both bone marrow and peripheral blood of MM. A significant correlation of NEAT1 expression between BM and PBMC was observed. Furthermore, based on the previous study of NEAT1, we hypothesized that NEAT1 may be used as an important biomarker to diagnose. Further more we supposed that if the expression of lncRNA NEAT1 may associated with Dex resistant in clinic, and it may be and predict the prognosis and treatment efficacy of multiple myeloma. Accordingly, the expression of NEAT1 was examined to analyze its relationship with MM development and prognosis and to explore the diagnostic value and clinical value of NEAT1 in MM.

## Materials and methods

### Patients and samples

The study cohort included 60 adult patients aged 45 years to 72 years with multiple myeloma diagnosed at the 2nd Affiliated Hospital of Harbin Medical University from 2015 to 2018 who were free from other malignant diseases. The diagnosis of multiple myeloma was confirmed by bone marrow examination which revealed a monoclonal plasma cell count over 10%. The diagnostic criteria, disease status and response to treatment were based on the criteria of the International Myeloma Working Group. In addition, bone marrow samples and blood samples were collected from 60 MM patients. The disease status of the post-treatment patients was based on the criteria of International Myeloma Working Group. In addition, the percentage of plasma cells in the patients achieving VGPR or CR after treatment was less than 5%.We also enrolled 21 bone marrow and blood samples from healthy donors as the control group whose bone marrow examinations revealed no abnormalities. All patients and healthy donors signed informed consent forms after the study had been thoroughly explained. [18].

### RNA extraction and reverse transcription

Bone marrow and peripheral blood mononuclear cells were isolated for this study. First, the bone marrow and peripheral blood samples were collected in 5ml tubes containing ethylenediaminetetraacetic acid (EDTA), preserved at 4 °C and processed within 4 h of collection. The bone marrow and blood samples were then centrifuged using lymphocyte separation medium, and mononuclear cells were collected. The isolated bone marrow and blood samples were stored at -80 °C until RNA extraction. RNAs was isolated by using the TRIzol protocol (Invitrogen). The extracted RNA was then treated with DNase (Promega) and the concentration was determined by spectrophotometric OD260 measurement. The integrity of the RNA was examined by 1.2% RNA denaturing agarose gel electrophoresis. The procedure was performed according to the manufacturer’s protocol (Applied Biosystems) [18].

### RT-PCR

PrimeScript RT Master Mix (Takara, Dalian, China) was used for RT-qPCR and cDNA reverse transcription in accordance with the product manual. SYBR Premix EX Taq™ II (Takara) was used for qPCR; the ABI 7500 system (Applied Biosystems, Waters, MA, USA) was used for sample loading. The detection procedures and reaction criteria were set and carried out with reference to the instructions provided with the test kit. Primers were as follows: NEAT1 forward, 5′-CTTCCTCCCTTTAACTTATCCATTCAC-3′; NEAT1 reverse, 5′-CTCTTCCTCCACCATTACCAACAATAC-3′. GAPDH was used as an internal reference with the following primers: GAPDH forward, 5′-GCACCGTCAAGGCTGAGAAC-3′; GADPH reverse, 5′-TGGTGAAGACGCCAGTGGA-3′. Tests on all samples were run in triplicate. The relative expression of RNA was computed based on the 2 − ΔΔCt method [30].

### Interphase fluorescence In-Situ hybridization (FISH)

Interphase FISH was performed in all cases on BM smears. We used probe to detect 13q14 deletion [del(13q14)] and p53 deletion. Fluorescent images were captured by epifluorescence microscope (DRMA2; Leica, Wetzlar, Germany) and CCD camera (JAI Company, London, UK) using appropriate filters. Two hundred nuclei were scored for each probe. BM cells samples of 10 cytogenetically normal individuals served as controls [31].

### Statistical analysis

The independent two samples t-test was used to compare the expression levels of NEAT1 in the different subgroups. Kruskal-Wallis H-test was used for multiple comparisons between subgroups. Analysis of correlation was performed using Pearson correlations or Pearson correlation coefficients. Receiver operating characteristic (ROC) analysis was used to evaluate the cut-off value. Survival curves were plotted using the Kaplan–Meier method. All statistical analyses were based on two-sided hypothesis tests with a significance level of p < 0.05. The analyses were performed using SPSS 17.0 software (IBM SPSS, Chicago, IL) and GraphPad Prism 5 (GraphPad Software, La Jolla, CA) [18].

## Result

### General clinical information

In total of 60 newly diagnosis MM patients were enrolled in the current study, include male 38 cases, female 22 cases, and the mean age is 50.61 ± 16.04 years old (range from 45 to 75). According to International staging system (ISS), all patients were divided into stage I: 10 cases, stage II: 18 cases and stage III: 32 cases. Isotype were distinguished in IgG: 36 cases, IgA: 16 cases and light chain type: 8 cases. According to PC percent of bone marrow, 35 patients were **≤** 50% and 25 patients were > 50%. At treatment, 38 patients received Bortezomib, dexamethasone (BD) regimen, and 22 patients received Bortezomib, Adriamycin, Dexamethasone (PAD) regimen. The chemotherapy regimen consisted of 2 to 8 cycles. In regard to cytogenetic factors, 36 patients were del(13q14) and 24 patients were non- del(13q14), 16 patients were p53 deletion and 44 patients were non-p53 deletion, 25 patients were 1q amplification and 35 patients were non-1q-amplification.

### Expression of NEAT1 in MM

**Fig. 1:**
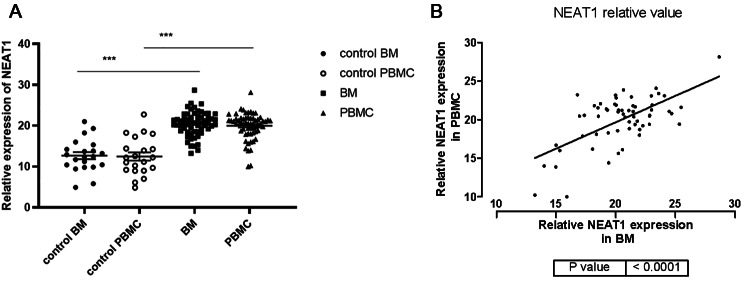
**A** The relative expression of NEAT1 in MM and healthy control group BM, PBMC (***P < 0.0001). **B** Pearson correlation analysis of NEAT1 expression level in MM BM and PBMC

The expression level of NEAT1 in bone marrow (BM) of 60 MM patients and 21 healthy donors was detected by real-time quantitative PCR to determine whether NEAT1 expression levels are higher in patients with MM. As shown in Fig. 1A, the significantly difference was found between MM patients and healthy controls. The BM of MM had a significantly increased NEAT1 expression compared with the level of healthy controls (20.432 ± 2.955 versus 12.663 ± 3.969, P < 0.01). To evaluated whether NEAT1 could serve as a circulating biomarker for MM patients, peripheral blood of MM patients and health controls were collected and analyzed to detect their relationship. Elevated NEAT1 levels were also observed in peripheral blood mononuclear cells (PBMCs). Also, NEAT1 expression level in PBMCs was positively associated with the expression of NEAT1 in BM (Fig. 1B r = 0.374, P < 0.01). It suggested that NEAT1 might serve as a promising circulatory biomarker for MM.

### Correlations between elevated NEAT1 and disease status in MM

An important attribute of good biomarkers is that they do not obviously change with clinical and laboratory parameters. As shown in Table 1, there was no significant difference in relative level of NEAT1 in PBMC between the 60 MM patients in terms of gender, age, immunoglobulin subtype, treatment regimens and while the correlation between NEAT1 expression and cytogenetic was estimated too weak to influence the usefulness of lncRNA NEAT1 as biomarkers for MM (all P > 0.05).

Regarding the relationship between the proportion of plasma cells in bone marrow and the relative expression level of NEAT1, we divided patients with the proportion of BM PC at or above 30% and below 30% into two groups, and found that the expression level of NEAT1 was statistically different between the two groups(P = 0.001). Pearson correlation analysis was conducted on the proportion of plasma cells in bone marrow and the expression level of NEAT1, and a positive correlation was found between the two(Pearson’ r = 0.61, P < 0.001)(Supplementary Figure S1).

In order to identify the clinical relevance of NEAT1 overexpression in MM, the correlation between NEAT1 expression and clinicopathological parameters were examined in BM and PB after all patients were divided into two groups of high NEAT1 expression(NEAT1-H) and low NEAT1 expression (NEAT1-L) in relation to the median. We found that among low lncRNA NEAT1 patients, the number of patients at ISS stages I, II, and III was 7(70.0%), 10(55.6%), and 12(37.5%) respectively; among NEAT1-H patients, the number of patients at different ISS stages was 3(30.0%), 8(44.4%), and 20(62.5%), respectively. This sames shows that high lncRNA NEAT1 patients have increased ISS stage compared to low lncRNA NEAT1 patients (P < 0.001) (Table 2).

**Table 1:** Clinical characteristics of the subjects in this study

Clinicopathologic features	cases	NEAT1 relative expression(mean ± SD)	P value
Age(years) ≤ 60 > 60	19 41	19.69 ± 3.28 20.09 ± 3.21	0.660
Gender M F	38 22	19.56 ± 3.45 20.66 ± 2.69	0.606
**Isotype** **IgG** **IgA** **light chain**	36 16 8	19.93 ± 3.1 20.11 ± 2.57 19.79 ± 3.51	0.975
**Regimen** **PAD** **BD**	22 38	19.90 ± 3.32 20.00 ± 3.19	0.913
**BM PC percent** **≤ 30%** **> 30%**	28 32	18.51 ± 3.26 21.23 ± 2.61	0.001
**Cytogenetic** **del(13q14)** **non- del(13q14)**	36 24	19.61 ± 3.30 20.49 ± 3.06	0.299
**Cytogenetic** **p53 deletion** **non- p53 deletion**	16 44	18.99 ± 3.99 20.31 ± 2.85	0.162
**Cytogenetic** **1q amplification** **non- 1q amplification**	25 35	20.26 ± 2.66 19.75 ± 3.58	0.546

**Table 2:** The relationship between ISS system and NEAT1

ISS system	cases	proportion	P value
**I** NEAT1-H NEAT1-L	10 3 7	30% 70%	< 0.01
**II** NEAT1-H NEAT1-L	18 8 10	44.4% 55.6%	
**III** NEAT1-H NEAT1-L	32 20 12	62.5% 37.5%	

### Diagnostic value of NEAT1 for MM

**Fig. 2:**
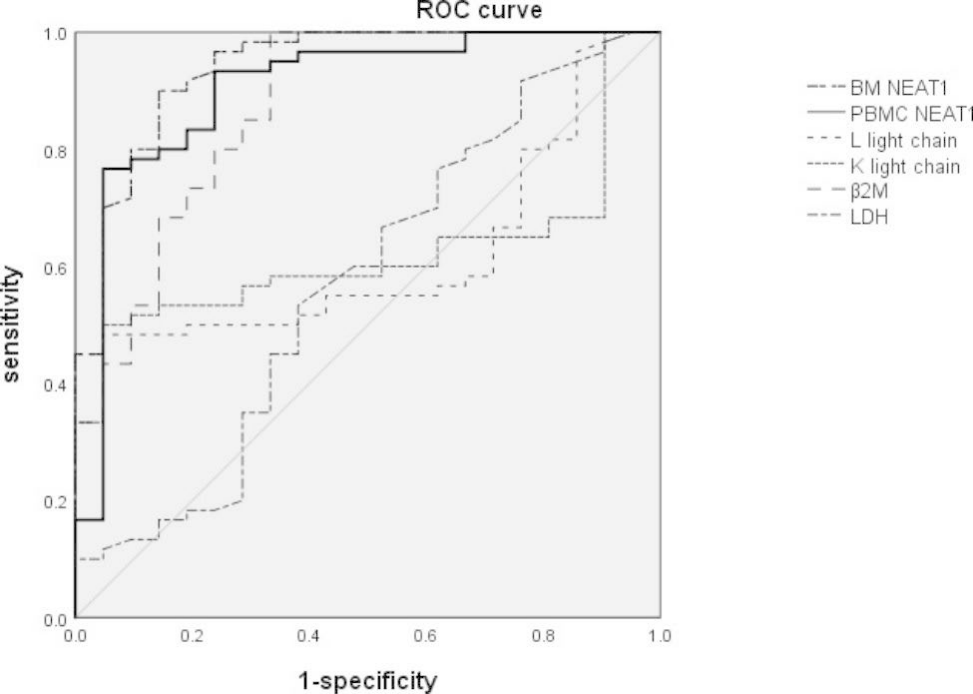
ROC curves of PBMC NEAT1, BM NEAT1, L light chain, K light chain, B2M and LDH activity for differentiating MM patients from healthy control group

To assess the potentiality of clinical application of NEAT1 in PBMCs, as we can seen in Fig. 2, ROC curves were employed to evaluate the diagnostic value of PBMC NEAT1, BM NEAT1, B2M, L light chain, K light chain and LDH in MM and healthy control groups. The AUC of PBMC NEAT1 was 0.922 (95% CI 0.857–0.988) for distinguishing MM patients and healthy control groups, and the related sensitivity and specificity were 80.0% and 71.0%. And the AUC of BM NEAT1 was 0.939 (95% CI 0.878–0.999). While, the AUC was 0.875 (95% CI 0.781–0.969) for β2M, 0.620 (95% CI 0.496–0.744) for L light chain, 0.620 (95% CI 0.499–0.741) for K light chain and 0.573 (95% CI 0.422–0.724) for LDH respectively. Compared MM and healthy controls, sensitivity was highest for NEAT1 (85.0%), while, specificity was highest for light chain (85.7%). The accuracy of NEAT1 alone for MM was 84.0%. Then, we combinate NEAT1 with L light chain, K light chain, β2M and LDH. It was shown that sensitivity was highest for a combination of NEAT1 and β2M, and specificity was highest for a combination of NEAT1 and light chain. These results indicated that NEAT1 could serve as a potential biomarker for MM(Table 3).

**Table 3:** Diagnostic efficacy of PBMC NEAT1, L light chain, K light chain, B2M and LDH in MM group as compared with that in control

Molecular marker	Sensitivity (%)	Specificity (%)	Accuracy (%)	Positive predictive values (%)	Negative predictive values (%)
PBMC NEAT1	85.0% (51/60)	80.9% (17/21))	84.0% (68/81)	92.7% (51/55)	65.4% (17/26)
L light chain	26.7% (16/60)	85.7% (18/21)	42.0% (34/81)	84.2% (16/19)	29.0% (18/62)
K light chain	25.0% (15/60)	85.7% (18/21)	40.7% (33/81)	83.3% (15/18)	28.6% (18/63)
β2M	48.3% (29/60)	95.2% (20/21)	60.5% (49/81)	96.7% (29/30)	39.2% (20/51)
LDH	13.3% (8/60)	76.2% (16/21)	28.4% (23/81)	61.5% (8/13)	23.5% (16/68)
NEAT1 + L light chain	86.7% (52/60)	76.2% (16/21)	84.0% (68/81)	91.2% (52/57)	66.7% (16/24)
NEAT1 + K light chain	86.7% (52/60)	76.2% (16/21)	84.0% (68/81)	91.2% (52/57)	66.7% (16/24)
NEAT1 + β2M	88.3% (53/60)	80.9% (17/21)	86.4% (70/81)	93.0% (53/57)	70.8% (17/24)
NEAT1 + LDH	75.0% (45/60)	76.2% (16/21)	75.3% (61/81)	90.0% (45/50)	51.6% (16/31)

### High expression of NEAT1 is associated with dex resistance

**Fig. 3:**
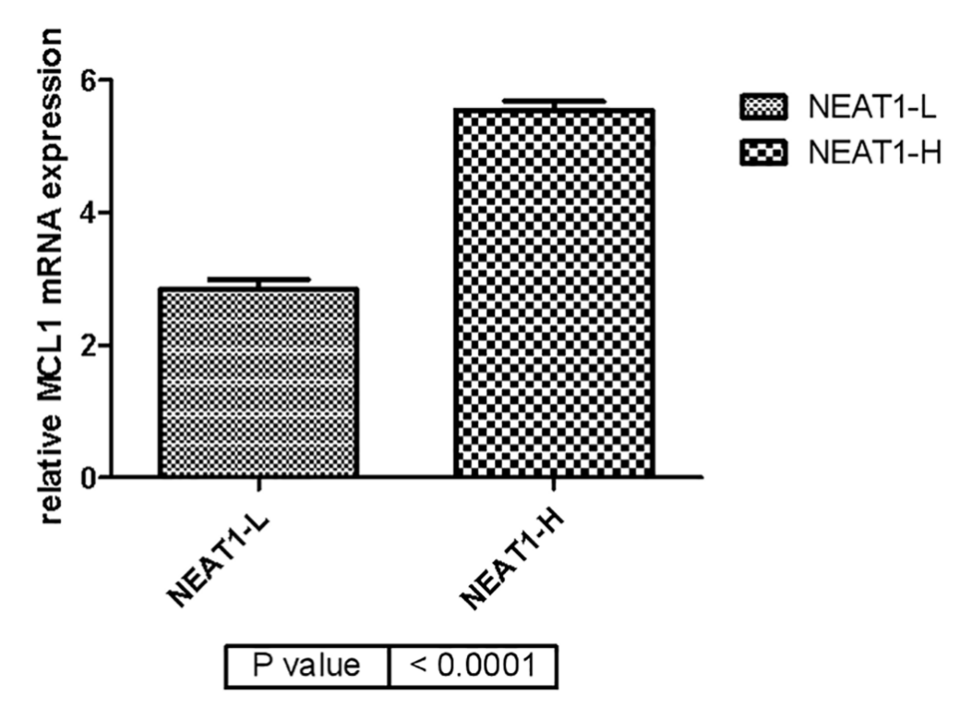
Comparison of relative MCL1 mRNA expression in low NEAT1 expression group and high NEAT1 expression group. Level of relative MCL1 mRNA expression in low NEAT1 expression group had a significant lower expression

At present, little is known about the role of lncRNAs into the circulating blood. To determine whether lncRNA NEAT1 can used as a biomarker for Dex resistance in MM, we observed the expression of MCL1 in all patients [32]. MCL1 is a potent anti-apoptotic protein that plays a critical role in cell survival and drug resistance in various cancers [33]. MCL1 constrained the growth of myeloma in vivo, and has been verified to be involved in the development of Dex resistance in MM cell lines [34]. According to the median NEAT1 expression level, we divided the patients into two groups: low NEAT1 expression group and high NEAT1 expression group. We examined the level of anti-apoptotic factor MCL1 in PBMCs of two groups, since Dex promotes MM cell death through induction of apoptosis. As shown in Fig. 3, we observed that patients with high NEAT1 expression had a significantly higher MCL1 expression. On the contrary, patients with low NEAT1 expression had a lower MCL1 expression. These showed that NEAT1 plays a certain predictive role on Dex resistance.

### Prognostic value of NEAT1 for MM

All patients received chemotherapy as BD or PAD, and we found there is no difference between NEAT1 level and regimens. Regarding course of treatment, after 2 courses, 43.5% CR rate and 56.6% VGPR rate were observed in low NEAT1 expression group, and 10.8% CR rate and 18.9% VGPR rate were observed in high NEAT1 expression group(Table 4). Compared to high NEAT1 expression group, the efficacy of chemotherapy is much better in low NEAT1 expression group. Further, we analyzed the curative effect after 4 courses in both groups, and the same result that a higher remission rate in low NEAT1 expression group was observed. These results indicate that lncRNA NEAT1 can predict the efficacy of chemotherapy in MM.

**Fig. 4:**
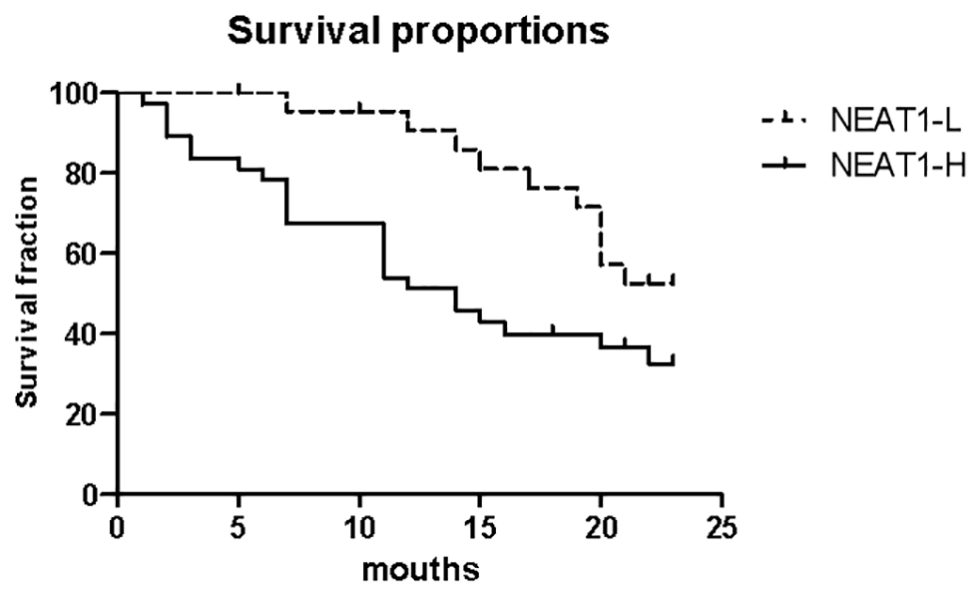
Correlation between peripheral blood NEAT1 expression and progression-free survival using Kaplan-Meier Estimates

Further, We assessed whether a direct correlation existed between NEAT1 expression and outcome in MM cases using PFS by Kaplan-Meier analysis. As shown in Fig. 4, the PFS 

of high NEAT1 expression group was significant lower than group low NEAT1 expression group, suggesting that in patients receiving BD or PAD therapy, high expression of NEAT1 was associated with worse survival. These results demonstrated that NEAT1 may play an important role in determining progression and the prognosis of patients with MM.

**Table 4:** The efficacy of CR or VGPR in different courses of treatment

	efficacy	value	
		Low NEAT1 expression(n = 23)	High NEAT1 expression(n = 37)
**2 courses of treatment**	≥ CR	10(43.5%)	4(10.8%)
	≥ VGPR	13(56.5%)	7(18.9%)
**4 courses of treatment**	≥ CR	15 (52.1%)	8(21.6%)
	≥ VGPR	18(78.3%)	11(45.2%)

## Dicussion

With the deepening understanding of the development of the disease and the diversification of treatment methods, the survival of patients with multiple myeloma has been significantly improved over the past 10 years, but the overall prognosis of MM patients is still poor. Dexamethasone (Dex) has been used clinically for decades as a first-line agent for MM therapy [3]. However, the use of Dex can cause serious side effects, and the resistance to Dex hampered its treatment [35], so a useful tool for diagnosis, prognosis and Dex resistance is urgently needed. In recent years, significant advances have been made in the cytogenetic and molecular characterization of multiple myeloma, but researchers continue to search for novel prognostic factors that will be useful tools for diagnosis and prognosis. Long non-coding RNA (lncRNA) is an emerging field of cancer research, involving many biological processes such as cell differentiation, apoptosis, epigenetic regulation of gene expression, and RNA attenuation [36]. It could be a new set of potential biomarkers and play a role in diagnosis, prognosis and treatment monitoring of diseases in different malignant diseases [37]. L. Sedlarikova have found some lncRNAs are unusually expressed in MM patients by microarray analysis, which revealed that NEAT1 expression levels in bone marrow (BM) is significantly higher in MM patients than health people [38]. However, what role this lncRNA plays in MM diagnosis and therapy remains unknown. In our study, we first elucidated the correlation between NEAT1 expression levels in peripheral blood (PB) and Dex resistance in clinic of MM patients.

We found that NEAT1 is dysregulated in the bone marrow of patients with multiple myeloma, but bone marrow puncture is an invasive procedure and inconvenient, that is awfully resisted by many patients. Because peripheral blood cells are derived from bone marrow, we examined the expression of NEAT1 in peripheral blood mononuclear cells (PBMCs) from healthy donors and MM patients. It was surprised that the expression level of NEAT1 was significantly higher in patients with multiple myeloma than in healthy donors. And interestingly, the expression level of NEAT1 in bone marrow was significantly correlated with peripheral blood mononuclear cells. This suggests that detecting the expression level of NEAT1 may be a more convenient method as far as a clinical test. In this study, we investigated the association of NEAT1 expression with clinicopathological characteristics and diagnosis value in MM. We showed for the first time that lncRNA NEAT1 in PBMCs was frequently upregulated in MM group than that in healthy control groups. In order to identify the clinical relevance of NEAT1 overexpression in MM, the correlation between NEAT1 expression and clinicopathological parameters were examined in BM and PB after all patients were divided into two groups of high NEAT1 expression(NEAT1-H) and low NEAT1 expression(NEAT1-L) in relation to the median. We found that high lncRNA NEAT1 patients have increased ISS stage compared to low lncRNA NEAT1 patients (P < 0.001) (Table 2). And we further found that NEAT1 positively associated with BM PC percent (Supplementary Figure S1). Our results also indicate that expression level of PBMCs lncRNA NEAT1 do not change appreciably with clinical parameters. The levels of NEAT1 in PBMCs do not change appreciably with age or gender, and they are also not associated with immunoglobulin subtype, treatment regimens (P > 0.05). Further, NEAT1expression levels is independent of the deletion of chromosome 13, p53 deletion, and 1q amplification. Our results further suggest that PB lncRNAs can be used as a diagnostic test for myeloma. Furthermore, the sensitivity of NEAT1 was 80.0% and the specificity was 71.0%, which indicate that NEAT1 has good sensitivity and high specificity. More important, our results show that the combination of NEAT1 and β2M provides optimal sensitivity for the detection of MM. Therefore, the relative expression of NEAT1 may prove to be a useful auxiliary test in the diagnosis of MM. Meanwhile, the PBMCs lncRNA expression level assessment is more reliable and easy to interpret than the traditional morphologic examination of medullary blast cells, and because it can be performed on sequential PB samples, lncRNA expression level appreciation may be more accurate than a single examination. Moreover, this technique appears to be useful clinically for an early and accurate appreciation of individual patients’ MM behavior during chemotherapy, which will possibly permit clinicians to better adapt therapeutic strategies.

Current first-line treatment in MM is based on proteasome inhibition and immunomodulation. With the development of high-throughput techniques, many novel agents about proteasome inhibition and immunomodulator had been researched for clinical treatment of MM, such as thalidomid and bortezomib [39]. However, Glucocorticoids (GCs) have been used clinically for decades as potent anti-inflammatory and immunosuppressive agents. In 1986, high-dose dexamethasone was added to the treatment guidelines for MM [40]. Recently, many studies had shown that the effects of proteasome inhibitors or immunosuppressive agents combined with dexamethasone [41], are much better than proteasome inhibitors or immunosuppressive agents alone. Simultaneously, it has been verified that dexamethasone can induce lymphocyte apoptosis, and it can inhibit the protein synthesis of tumor cells when combined with cytotoxic drugs, promote protein decomposition and improve the efficacy of cytotoxic substances [42]. Owing to its anti-inflammatory and immune-suppressive actions, Dex as a key front-line chemotherapeutic for B-cell malignant MM, participate in multi-drug chemotherapy regimens and play a critical role in chemotherapy [40]. Nonetheless, their use is severely hampered by the risk of developing side effects and the occurrence of Dex resistance, such as poor wound healing, increased risk for infections, osteoporosis, Cardiovascular complications and so on [43]. On account of chemotherapy requires large doses of dexamethasone and most patients require long-term maintenance therapy, dexamethasone resistance and side effects are unavoidable. And due to chemotherapy is usually combined, it is hard to judge clinically whether it exists Dex resistance, leading to side effects which are the cause of death at times. Therefore, we urgently need a biomarker to determine Dex resistance and adjust the treatment strategy effectively.

MCL1 is a potent anti-apoptotic protein that plays a critical role in cell survival and drug resistance in various cancers. It can help tumor cells escape drug attacks and continue to grow [44]. At the same time, VEGF-induced MM cell proliferation and survival are also mediated by MCL1. Elevated expression levels of MCL1 prevents cancer cells from initiating apoptosis in the face of many intrinsic tumor-suppressing pathways and extrinsic therapeutic treatments aimed at controlling tumorigenesis [45]. Recently, MCL1 has been a potent anti-apoptotic protein that plays a critical role in cell survival and drug resistance in various cancers. What is more, targeting MCL1 also constrained the growth of myeloma in vivo, which is pivotal for maintaining survival of most myelomas, and it should be prioritized for targeting in the clinic. Meanwhile, MCL1 participate in Dex resistance in MM1S and MM1R cell lines. Researchers have found that knockdown of MCL1 significantly decreased the anti-apoptosis and proliferation ability of NEAT1 overexpressing MM1S cells, and suggest that MCL1 mediated the effect of NEAT1 on MM cells DEX resistance [46]. In the current study, the median of the expression level of MCL1 was estimated to analysis the role of NEAT1 contributing to the Dex resistance and it was obtained that up-regulated expression of NEAT1 in patients is associated with Dex resistance. It suggested that NEAT1 can be used as a biomarker for Dex resistance in MM. For further confirmation, the correlation between expression of NEAT1 and the efficacy of chemotherapy in MM was certified. As for treatment, upregulated expression of NEAT1 indicated worse survival in patients. These all indicated the possibility of NEAT1in judging Dex resistance and prognosis, but it still requires confirmation in further studies.

In summary, we have shown that the expression of NEAT1 in PBMC is independent of age, gender, disease stage, BM PC percentage, myeloma protein and cytogenetic factors, suggesting that lncRNA in peripheral blood can be used as a diagnostic test for myeloma. Meanwhile, NEAT1 is associated with the expression of MCL1 which is a potent anti-apoptotic protein including Dex resistance. These findings indicate that NEAT1 is an important molecular marker for predicting prognosis to overcome DEX resistance in MM therapy. Our study indicated that NEAT1 might act as a promoting factor in the development of MM and could be a diagnostic factor, therapeutic effect evaluator and prognostic indicator in the prognosis of MM. The lncRNA has a significant value during the chemotherapy and evaluation the therapeutic strategies. However, the underlying mechanism of NEAT1 in Dex resistance is still unclear, its role in the pathogenesis of this disease should be examined further.

## Electronic supplementary material

Below is the link to the electronic supplementary material.

Supplementary Material 1

## Data Availability

The datasets used and/or analysed during the current study are available from the corresponding author on reasonable request.
