# Supplementary material for: Expression of NEAT1 can be used as a predictor for Dex resistance in multiple myeloma patients
Source: BMC Cancer. 2023 Jul 5;23:630. doi: 10.1186/s12885-023-11084-x (PMC10320976; doi:10.1186/s12885-023-11084-x)
Supplement: Supplementary file 1 — Supplementary Material 1 [file 12885_2023_11084_MOESM1_ESM.docx]

**Supplementary Figure**

**
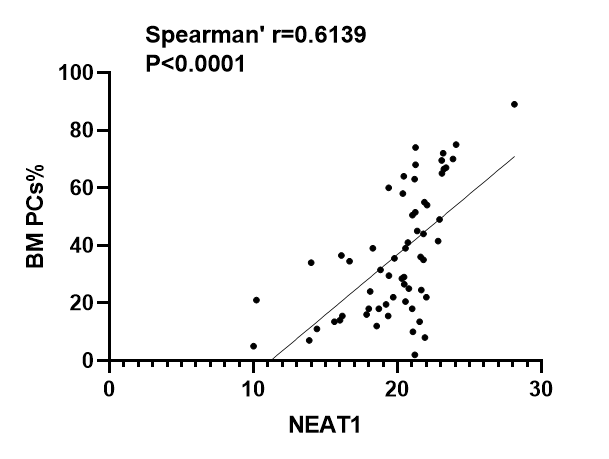
**

*Supplementary Figure S1. Correlation between peripheral blood NEAT1 expression and BM PCs% using Pearson correlation analysis.*
